# The Biology of Malignant Mesothelioma and the Relevance of Preclinical Models

**DOI:** 10.3389/fonc.2020.00388

**Published:** 2020-03-25

**Authors:** Christophe Blanquart, Marie-Claude Jaurand, Didier Jean

**Affiliations:** ^1^Université de Nantes, CNRS, INSERM, CRCINA, Nantes, France; ^2^Labex IGO, Immunology Graft Oncology, Nantes, France; ^3^Centre de Recherche des Cordeliers, Inserm, Sorbonne Université, Université de Paris, Functional Genomics of Solid Tumors Laboratory, Paris, France

**Keywords:** thoracic cancer, mesothelioma, molecular characteristics, tumor heterogeneity, preclinical models, cell models, animal models

## Abstract

Malignant mesothelioma (MM), especially its more frequent form, malignant pleural mesothelioma (MPM), is a devastating thoracic cancer with limited therapeutic options. Recently, clinical trials that used immunotherapy strategies have yielded promising results, but the benefits are restricted to a limited number of patients. To develop new therapeutic strategies and define predictors of treatment response to existing therapy, better knowledge of the cellular and molecular mechanisms of MM tumors and sound preclinical models are needed. This review aims to provide an overview of our present knowledge and issues on both subjects. MM shows a complex pattern of molecular changes, including genetic, chromosomic, and epigenetic alterations. MM is also a heterogeneous cancer. The recently described molecular classifications for MPM could better consider inter-tumor heterogeneity, while histo-molecular gradients are an interesting way to consider both intra- and inter-tumor heterogeneities. Classical preclinical models are based on use of MM cell lines in culture or implanted in rodents, i.e., xenografts in immunosuppressed mice or isografts in syngeneic rodents to assess the anti-tumor immune response. Recent developments are tumoroids, patient-derived xenografts (PDX), xenografts in humanized mice, and genetically modified mice (GEM) that carry mutations identified in human MM tumor cells. Multicellular tumor spheroids are an interesting *in vitro* model to reduce animal experimentation; they are more accessible than tumoroids. They could be relevant, especially if they are co-cultured with stromal and immune cells to partially reproduce the human microenvironment. Even if preclinical models have allowed for major advances, they show several limitations: (i) the anatomical and biological tumor microenvironments are incompletely reproduced; (ii) the intra-tumor heterogeneity and immunological contexts are not fully reconstructed; and (iii) the inter-tumor heterogeneity is insufficiently considered. Given that these limitations vary according to the models, preclinical models must be carefully selected depending on the objectives of the experiments. New approaches, such as organ-on-a-chip technologies or *in silico* biological systems, should be explored in MM research. More pertinent cell models, based on our knowledge on mesothelial carcinogenesis and considering MM heterogeneity, need to be developed. These endeavors are mandatory to implement efficient precision medicine for MM.

## Introduction

The therapeutic options for malignant mesothelioma (MM) are limited, especially for the most common form of mesothelioma, malignant pleural mesothelioma (MPM). Current MPM chemotherapy is based on intravenous injections of pemetrexed (PMTX) and cisplatin or carboplatin. Recently, this basic treatment has been improved by the addition of vascular endothelial growth factor (VEGF) antibodies (bevacizumab), where the overall survival of patients receiving PMTX, cisplatin, and bevacizumab was significantly enhanced (MAPS study) (1). Furthermore, immunotherapy-based strategies are currently becoming attractive therapeutic options, and several clinical trials have recently been performed. A phase II study using monoclonal antibodies against cytotoxic T-lymphocyte antigen 4 (CTLA4; tremelimumab) in patients showing progression of the disease after first-line treatment yielded encouraging results, but it was performed in a small number of patients (2). Another phase II study (DETERMINE) investigated the effect of tremelimumab in patients whose disease had progressed after one or two systemic treatments. There were no benefits, but the safety profile was acceptable (3). A more recent phase 2 study (IFCT-1501 MAPS2) reported the use of immune control checkpoint inhibitors, programmed cell death protein 1 (PD-1; nivolumab) and cytotoxic T-lymphocyte-associated protein 4 (CTLA4; ipilimumab), alone or in combination. The results showed objective anti-tumor responses and a significant increase in survival without progression and global survival (4). Clinical trials for cell-based immunotherapy using dendritic cells or chimeric antigen receptor T (CAR-T) cells have also yielded promising results (5–10).

In the future, new clinical trials will be developed that utilize novel anti-cancer compounds or immunological modulators in association with chemotherapies or in combination with immunological approaches. The efficiency of current treatments are dependent on the integrity of metabolic pathways and DNA repair mechanisms that account for resistance mechanisms. Overall, therapy improvements require better knowledge of the state of the cell regulatory pathways. In addition, immunotherapies need sound knowledge about the immunological status of the tumor. To date, molecular data are not ordinarily used to assist therapeutic decisions, and thus there is an urgent need for their use in translational medicine. To reach these goals, two different fields must be investigated: (i) the cellular and molecular status of MM tumors, regarding mutations, alterations in regulatory pathways, and the microenvironment landscape, and (ii) the methodology of preclinical assays to soundly test specific anti-tumor agents. The aim of this review is to provide an update on our present knowledge and issues on these subjects and to provide perspectives for advancements in MM treatment.

## The Biology of Malignant Mesothelioma

Malignant mesothelioma are heterogeneous tumors that show a complex pattern of molecular changes, including genetic, chromosomic, and epigenetic alterations, all of which should be considered to model this pathology. Of all the MM types defined by tumor location, MPM has the best described molecular alterations and heterogeneity, and thus we will focus on it. Notably, recent integrative multi-omics analysis as well as next generation sequencing (NGS) studies on malignant peritoneal mesothelioma (MPeM) showed similarities to MPM in terms of molecular alterations, even though some alterations, such as *ALK* rearrangement, are only found in MPeM (11–13).

### Molecular Alterations

Recent NGS studies identified a low mutation burden in MPM compared to other adult solid tumors (14). However, this mutation burden could be underestimated by classical NGS analyses, which focus on the detection of changes at the nucleotide level. Early karyotyping analyses and molecular cytogenetic techniques, such as comparative genomic hybridization (CGH) and single nucleotide polymorphism (SNP) arrays, showed that MPM is characterized by numerous chromosomal abnormalities, including abundant numeric and structural chromosome changes and recurrent alterations in specific chromosome regions (15). More recently, a combination of high-density array-CGH with targeted NGS demonstrated the presence of chromothripsis in the 3p21 region, which includes the *BAP1* gene (16). Chromothripsis and also chromoplexy were confirmed on several other chromosome regions in MPM using mate-pair sequencing (17). These numerous inter- or intra-chromosomal rearrangements may result in the disruption of tumor suppressor genes (TSG) as well as the amplification of oncogenes or fusion genes that can drive carcinogenesis.

The mutated genes in MPM are essentially TSG that are inactivated by several mechanisms, including single nucleotide variants, copy number losses, gene fusions, and splicing alterations (14, 18). The only recurrent oncogenic mutation was identified in the promoter of *TERT*, which encodes telomerase, the essential enzyme that maintains the length of the telomeres (19). The most frequently altered TSG are *CDKN2A, BAP1*, and *NF2*, and to a lesser extent *TP53, SETD2*, and *LATS2*. All of the other mutated genes show <3% somatic mutation (14, 18). Germline mutations that predispose to MPM were first identified in *BAP1*, but two recent studies also highlighted germline mutations in several other genes that are less common than in *BAP1*. They are mainly involved in cell-cycle, chromatin regulation and DNA repair (20–24). Up to 7% of MPM patients may have germline mutations, but experimental validations are needed to confirm that some of these genes are MPM susceptibility genes (like *BAP1*).

The epigenomic landscape of MPM has also been investigated, albeit to a lesser extent. A microarray-based methylome analysis demonstrated that MPM has specific patterns of gene methylation compared to normal pleura or other tumors (25, 26). The contribution of DNA methylation to mesothelial carcinogenesis has been clearly established, notably by the downregulation of TSG expression (27). The mechanisms for epigenetic regulation in MPM were principally studied in the context of *BAP1* inactivation; they highlighted the role of polycomb repressive complex 2 (PRC2) and histone methyltransferase (28). Other studies also emphasized the involvement of non-coding RNA such as micro-RNA (miRNA) or long non-coding RNA (lncRNA), both of which are deregulated in MPM, in carcinogenesis (29–31).

Altogether, these molecular alterations lead to changes in gene expression and deregulation of several biomolecular pathways, including signaling pathways such as Hippo or the PI3K/AKT/mTOR pathways, the cell cycle and apoptosis, among others (32). The implication for therapy from all these molecular changes has been recently reviewed (33).

### Mesothelioma Heterogeneity

Like most adult solid tumors, MM is a heterogeneous cancer with high variability among patients. Hence, the development of experimental models must consider this heterogeneity. Histology defines three major types of MM: epithelioid, the most frequent histological subtype; sarcomatoid, with the worst prognosis; and biphasic, which is a mixture of the two previous morphologies. Histological subtypes within these three types have been defined (34). The histological classification only partially captures the tumor heterogeneity observed at both the molecular and clinical levels (35). Large-scale omics and NGS studies have demonstrated MPM heterogeneity at the molecular level that goes beyond the histological classification (14, 18, 36). The first MPM molecular classification, related to histological types and survival, proposed two tumor subtypes by clustering transcriptomic data (36). A new subtype with a poor prognosis and characterized by a double mutation in the TSG *NF2* and *LATS2*, both of which are involved in the Hippo signaling pathway, was identified by coupling genetic and transcriptomic analysis (37). Other studies have proposed classifications into four subtypes that are also related to prognosis and partially to genetic alterations (14, 18, 38). Interestingly, a meta-analysis that compared the subtypes obtained by clustering from several transcriptomic data sets showed that only the most extreme subtypes, which represent the “pure” epithelioid and sarcomatoid phenotypes, are found in all datasets. These findings suggest that intermediate subtypes might only reflect divisions of a continuum (38, 39). Based on these results, histo-molecular gradients obtained by a signal deconvolution method on transcriptomic data were proposed to consider MPM inter-tumor heterogeneity as well as intra-tumor heterogeneity. These histo-molecular gradients determine the variable proportion of epithelioid and sarcomatoid tumor cell contingents in tumor samples. They also have a strong prognostic value and may be of interest for guiding therapeutic strategies (38, 39). Another recent publication further sustained that MPM heterogeneity is better described by a continuum (40).

Intra-tumor heterogeneity is still partially described in MPM, in part due to the use of omics approaches only in bulk tumor samples. MPM is likely a polyclonal tumor that comprises multiple subclones with variable cellular prevalence (41, 42). To better define the polyclonal tumor origin and understand the tumor evolution of mesothelioma, further studies are required in a larger number of tumor samples. Several studies also highlighted the presence of cancer stem cells in MPM (35). In MPM, heterogeneity is not limited to tumor cells; the tumor microenvironment is also distinct from one patient to another in terms of type and number of stromal and immune cells that infiltrate the tumors (43). Immune signatures are linked to the patients' outcome (44). Spatial heterogeneity of the somatic mutations of cancer cells, as well as the immune microenvironment, was highlighted by studying tumor samples at different anatomic sites (45). In this complex context, the use of the emergent “single cell” approaches will be helpful in providing an accurate characterization of tumor and stromal cell heterogeneity and should contribute to a breakthrough in knowledge about intra-tumor heterogeneity. Besides the inter- and intra-tumor heterogeneity of tumor cells, the evolutionary features of tumors need to be considered to establish a classification that is clinically relevant (46).

## Preclinical Models

**Figure 1:**
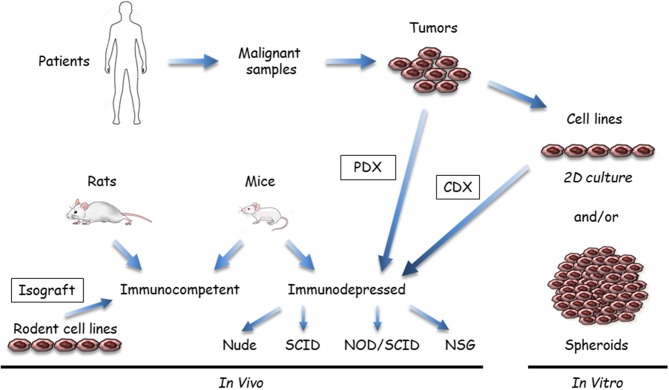
Available malignant mesothelioma (MM) preclinical models that use tumor samples or cell lines. Malignant samples are tumor fragments or more often MM cells obtained after tissue dissociation; these samples are used *in vitro* or transplanted/inoculated in immunosuppressed or immuno-compatible rodents, almost exclusively mice. *In vitro*, two-dimensional (2D) cultures or three dimensional (3D) spheroids can be grown from tumor tissue samples. *In vivo*, MM tumor cells in culture are inoculated either subcutaneously or orthotopically (intracavitary, in the pleura or peritoneum) to generate cell-derived xenograft (CDX) or isograft. Tumor fragments can be also engrafted into immunosuppressed mice (patient-derived xenograft [PDX]). Immunocompetent models mainly comprise syngeneic rodent models. Human immunocompetent models can be obtained using NOD-*scid IL2R*γ^*null*^ (NSG) mice.

**Figure 2:**
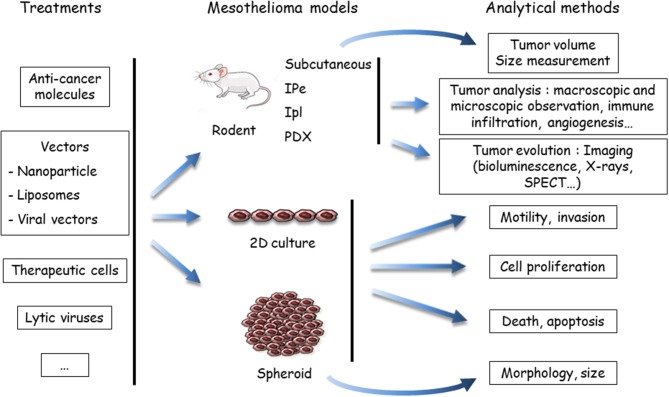
Evaluation of treatments using preclinical models of malignant mesothelioma (MM). Anti-cancer agents are delivered to the MM models, either in a native, or vectorized form (in nanoparticles, liposomes, or viruses). MM cells may be labeled or engineered to allow one to determine tumor growth by imaging methods, mostly bioluminescence. To determine the host response, the analytical method depends on the MM model. *In vivo* endpoints comprise measuring the tumor volume, quantifying and identifying tumor cells in the pleural effusion or ascites (if produced), or measuring others parameters (angiogenesis, immune infiltration, etc.). *In vitro*, the endpoints are cell viability, type of death, proliferation, motility, invasion, morphology, and volume (spheroids). Both cell and mouse models have been applied to test the efficiency of drugs in mono- or multimodality chemotherapies, immunotherapies and target therapies, oncovirotherapies, or cell and gene therapies. SPECT, single photon emission computed tomography; IPe, intra-pleural; Ipl: intra-peritoneal; PDX, patient-derived xenograft.

In this section, we will focus on preclinical models that are useful for chemotherapy, targeted therapy, or immunotherapy rather than for surgery or radiotherapy, even though those therapies have a place in the treatment of patients. The efficiency of anti-cancer compounds to treat MM patients has been tested using large variety of so-called preclinical MM models. These systems are based on use of human or mammalian MM samples, i.e., xenografts in immunosuppressed mice or isografts in syngeneic rodents. Multiple combinations have been developed based on the nature of the malignant sample (cells or tumor tissue), the recipient (rats or mice), the anti-cancer agent (anti-cancer drug, lytic virus, therapeutic cells sur as dendritic or CAR-T cells, etc.), the agent vector (if any), the method to implant tumor cells, and the analytical method. These models do not exactly reproduce human MM, but they are surrogates for a proof of concept. Preclinical model options are synthetized in Figures 1, 2, and the main points are described below:

(i) Samples and recipients (Figure 1): Several MM samples are used for preclinical studies. Tumor fragments, pleural liquid, and ascites can be collected from patients. Commercial MM cell lines are available from different companies, but primary MM cell lines are a better model, as extensively discussed in the *in vitro* models section (see below). Cell models in culture mostly comprise two dimensional (2D) MM cells or three dimensional (3D) multicellular tumor spheroids (MCTS). These cell models are generally monoculture, but new developments include the introduction of stromal and immune cells to better recapitulate the tumor microenvironment. *In vivo* models are based on the injection of MM cells in subcutaneous (SC) or orthotopic (intrapleural [IPl] or intraperitoneal [IPe]) sites in relevant rodents, mainly mice. Fresh MM tissue samples can be also grown as tumoroids (tumor-derived organoids) in culture or xenografted in immunosuppressed mice as patient-derived xenografts (PDX). Regarding the heterogeneity of human MM, it is important to work with well-characterized MM, particularly when drugs have been designed to target a single protein or a specific pathway. With our developing knowledge of the MM biology, it appears that multiple samples would have to be used. Furthermore, MM classification according to data arising from multi-omic studies (12, 14, 18, 36, 38) might help to define key alterations representative of molecular subtypes of MM and limit the studies on representative samples.

(ii) Anti-cancer compounds (Figure 2): These compounds are intended for chemotherapy, target therapy, immunotherapy, gene therapy, or oncovirotherapy because MM is a compartmentalized tumor with accessibility for *in vivo* local delivery (47, 48). They are used alone or in combination with other compounds, and as a single molecule or vectorized. Preclinical studies on the chemotherapeutic agent PMTX illustrated this diversity. The effects of PMTX have been investigated in association with several anti-tumor agents (anti-tubulin, gemcitabine, cisplatin, anti-thymidylate synthase, RNA interference [RNAi] embedded in liposomes, miRNA expressed in adenovirus vector, etc.) to determine a potential synergistic effect (49–55). Liposomal PMTX formulations have been tested in an orthotopic mouse model (56). Due to the diversity of the assays, it is difficult to compare their predictability.

(iii) Analytical methods (Figure 2): The endpoints for *in vitro* assays comprise the determination of cell proliferation, cell death, motility and invasive properties, and spheroid state (morphology and volume). *In vivo* tumor analyses involve macroscopic and microscopic observations and evaluation of immune infiltration and angiogenesis. The key point is to monitor tumor evolution, especially for orthotropic tumor grafts. Different analytical methods have been developed for *in situ* tumor visualization. Firefly luciferase (*luc*)-engineered cells can be detected by a non-invasive bioluminescence imaging method, as in rats injected with *luc*-MM cells in the pleural cavity (57). However, data have shown that magnetic resonance imaging (MRI) is a more reliable method for MPM tumor burden measurement compared to bioluminescence (57). Computed tomography scanning may be also of interest, as shown with a lung cancer cell line in mice (58). Tumor lesions and the localization of epidermal growth factor receptor (HER) were visualized with single photon emission computed tomography (SPECT) and MRI in an orthotopic MM model with radiolabeled specific antibodies (59). Bioluminescence of *luc*-expressed MM remains the most common strategy to monitor tumor development in orthotopic models. These *in vivo* imaging methods require specific equipment and facilities and, for bioluminescence detection, the genetic modification of tumor cells with the *luc* gene. The introduction of an exogenous gene might have an impact on cell mechanisms and immune response.

In the following subsections, we detail the present and ongoing models, with a focus on their interest, limitations, and impacts to assess emerging therapies. The advantages and limitations of the mesothelioma preclinical models are presented in Table 1.

**Table 1:** Advantages and limitations of the different models of mesothelioma.

**Model**	**Advantages**	**Limits**
Monolayer cells	Easy to obtain Suitable for high-throughput screening	Clonal selection in culture Response to therapies is poorly representative No microenvironment
Multicellular tumor spheroids (MCTS)	Easy to obtain Three-dimensional structure constraints Suitable for high-throughput screening Common features with tumors *in situ*	Not heterogeneous Partial and artificial microenvironment
Cell-derived xenograft (CDX)	Source of tumor cells easy to obtain (cell lines in culture) Useful for evaluation of targeted strategies	Not heterogeneous No microenvironment Immunodeficient context Response to therapies is poorly representative
Patient-derived xenograft (PDX)	Tumors with characteristics of the of patients (heterogeneity, microenvironment) Representative response to therapies	Availability of the tumor Time to obtain the tumor Not suitable for immunotherapy evaluation
Humanized model (NSG)	Human immune system Evaluation of immunotherapy possible	Time to obtain tumor Cost Graft vs. host response

### *In vitro* Models

MM cell lines have been widely used to study MM pathogenesis and evaluate the activity of numerous anti-cancer agents. The first MM cell lines were established in 1982 from the abdominal fluid of a patient (60). In 1987, a MM cell line was established from a surgical sample of malignant pleura, namely H-Meso-1 (61). Since that study, numerous cell lines have been established from samples of patients by different groups to constitute local biocollection. Some of these collections have been extensively characterized (19, 36, 37, 62–66), as well as 21 cell lines in the Genomics of Drug Sensitivity in Cancer (GDSC) database (https://www.cancerrxgene.org/celllines). MPM cell lines present common characteristics with regard to tumors and might lead to the identification of new biomarkers (62, 67, 68). One study discussed the limits of these cell models. The authors found strong molecular differences between primary and commercial cell lines (67), mainly due to a high number of divisions after their establishment, and thus an increased risk of new karyotypic changes. These models remain interesting for screening and preliminary investigations. Primary tumor cells represent an intriguing alternative because they share similar molecular characteristic with the primary tumor, even though they show a reduction of subclonal diversity (15, 42, 67). However, the necessity to perform studies before 6–10 passages limits the number of experiments. The most appropriate strategy would probably be to conduct large screening studies on cancer cell lines and then confirm the findings with primary cancer cells. The results obtained with cell lines should be confirmed on samples from patients (if applicable).

### *In vivo* Models

**Figure 3:**
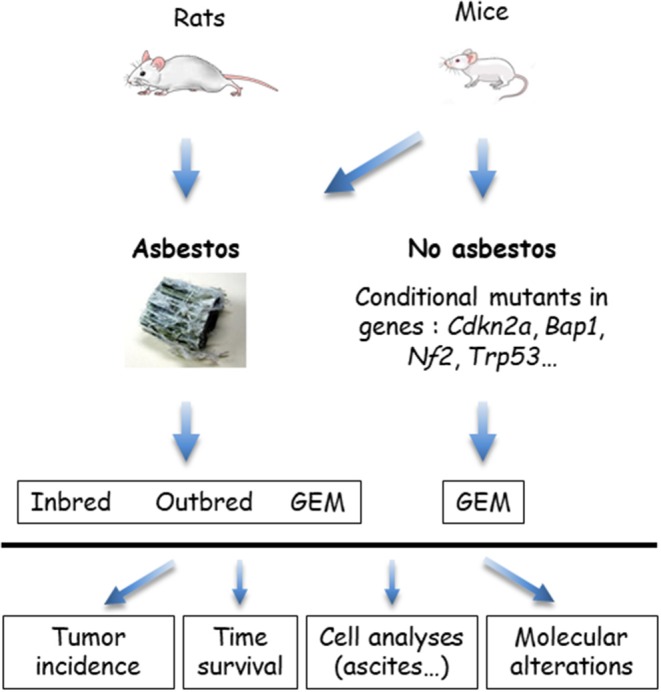
*In vivo* models of malignant mesothelioma (MM) in asbestos-treated rodents and genetically modified mice (GEM). MM can be obtained from wild type (WT) rats or mice, exposed to asbestos, or GEM. Asbestos-induced MM models are generated by the injection of asbestos fibers intracavitary, in the pleura or peritoneum, mostly peritoneal in mice. Conditional mutant mice are obtained by engineering the major genes altered in MM. Asbestos-recipient animals and GEM may be investigated for tumor incidence, survival, quantitative and qualitative analyses of cells in ascites or pleural fluid, and molecular alterations in tumors.

The *in vivo* models that use wild type rodents and genetically modified mice (GEM) are summarized in Figure 3. GEM have been generated to obtain “spontaneous” MM, without exposure to asbestos fibers, by heterozygous (htz) or homozygous (hom) conditional mutation of *Ink4a* and/or *Nf2* and/or *Trp53*, or by IPl/IPe injection of Ad*Cre* to mimic the human condition (69). In this system, the rate of MPM is dependent on the type of inactivated genes; high rates occur with at least two hom genes, including *Trp53*. Survival generally exceeds 30–50 weeks, although shorter survivals occur in a few situations with hom/hom combinations (70). Similar results were recently reported by inactivating *Ink4a, Nf2*, and *Bap1* (71). The generated MM express a similar morphology to human MM, with a proportion of each histological type depending on the modified genes.

MM have also been generated in several types of GEM exposed to asbestos fibers IPe injections. Experimental cancers induced in animals by the responsible carcinogen better reflect the natural history of these cancers. They are particularly relevant for the coupled asbestos-MM condition, given that the large majority of human MM cases are linked to asbestos exposure. With regard to conditional mutant mice, it takes several months to more than 1 year before the development of a MM. While the morphological features are reproduced, the sarcomatoid MM subtype most frequently forms, contrary to what is observed in humans (70).

These sophisticated models have been mainly used for mechanistic purposes, with a focus on the molecular mechanism of MM formation or mechanism of action of asbestos fibers. According to our knowledge, GEM with mutated genes that are relevant to human MM have not been used to test the effects of drugs. However, models of colorectal, non-small-cell lung, and pancreatic cancers have been used to predict therapeutic responses (72, 73). Although these models are physiologically different from humans, GEM mice form tumors that carry relevant gene changes, show histological similarities, and should allow one to perform tests in an immunocompetent environment. However, there are several biological and technical pitfalls. For instance, the tumor evolution can differ among mice, with the possible occurrence of metastases, other types of tumors may be generated, and the physiological differences between mice and human may bias the predictive value of the assays. Otherwise, the complexity of these models makes it difficult to produce homogeneous data from a rather small number of mice, to detect the tumor without autopsy, to follow its evolution, and to determine the right time of its development to establish the planed protocol.

### Specific *In vivo* Models for Immune Therapies

Recent successes were obtained with the use of immune checkpoint inhibitors in clinical trials (2, 4, 74–76). However, the response rate remains limited and, therefore, the objectives are now to extend the benefit of these approaches to a large number of patients. Preclinical studies performed in appropriate *in vivo* models are mandatory to obtain relevant results and achieve this objective. The first criterion is the presence of a completely functional immune system, a factor that excludes xenograft models that use human tumor cells. Several immunocompetent models of MM have been developed in rodents using cell lines obtained after inoculation of asbestos fibers in the peritoneal cavity (Table 2).

**Table 2:** The main immunocompetent rodent models of mesothelioma.

**Rodent strain**	**Cell lines**	**References**
C57Bl/6 mice	AK7 AE17	(77) (78)
Balb/C mice	AB1 AB12	(79)
CBA/J mice	AC29	(79)
Fischer F344 rats	IL45 M5-T2, F5-T1, F4-T2, M5-T1	(80) (81) (82)

C57Bl/6 mice models with murine MM cell lines have been extensively used to evaluate immunotherapeutic approaches. The most utilized cell lines are AE17 and AK7 (77, 78). These cells have been modified, including exogenous expression of Ova as a neo-antigen, to increase their immunogenicity and evaluate a strategy to improve the anti-tumor immune response (78). Models of MM on the Balb/C genetic background, using AB1 and AB12 cells lines, are also available. CBA/J mice injected with AC29 cells can also be used as an immunocompetent model of MM; however, they have been exploited less than the other previously cited models (79). The main injection route to induce MM is IPe. Immunocompetent IPl models of MM have also been developed, but they are not frequently used due to the procedure required to access to pleural space. In both cases, tumor development is monitored by the previously mentioned imaging methods. SC injections are also used to overcome practical concerns, such as measurement of the tumor size and intra-tumor injection of therapeutics, but SC location is far from the pathophysiological context.

A MM model in Fischer F344 rats following IPl injection of IL45 cell line has also been described (80). With regard to rodent models, IL45 cells expressing *luc* was used to improve the monitoring of tumor development (83). Recently, models of MPeM in Fischer F344 rats have been developed (81). This effort generated several MM cell lines with distinct aggressiveness. Depending on the cell lines used, the immune infiltrate was different (with or without lymphocyte and/or macrophage infiltration of the tumors) (82). Therefore, the efficacy of immunotherapy approaches could be evaluated using this model in the appropriate immune context.

### Xenograft PDX and Humanized Models

The previously mentioned rodent models allow one to evaluate therapeutic strategies in a living organism with several constraints: elimination, diffusion in the tissue, bio-distribution, and toxicity. These models were particularly used for the evaluation of target therapies using inhibitors of histone deacetylases or signal pathways such as Hippo or focal adhesion kinase (FAK) (84–86), anti-mesothelin or anti-podoplanin antibodies (87–92), or CAR-T cells (5, 93, 94). However, they showed major limitations: (i) differences compared to humans in the immune system and metabolism of chemotherapeutic agents for syngeneic rodent models; and (ii) the use of a human cell line to induce tumors, which does not reflect human intra-tumor heterogeneity, in the context of immunodeficient mice (xenografts). In order to improve the relevance of rodent models, PDX models were developed and they implies implanting a tumor fragment from a patient into an immunodeficient mouse. For MM, the implantation was only heterotopic. Mouse strains with different levels of immunodeficiency can be used depending on the objective of the experiment (Nude, SCID, NOD-SCID, or NOD-*scid IL2R*γ^*null*^ [NSG]) (95). The first description of PDX models of MM was in 1980 (96). Tumors from three patients were transplanted into nude mice but only two tumors grew. Recently, SC implantation of tumors from 50 patients with MPM was evaluated in nude mice (97). This methodology maintains the heterogeneity of the tumor and its microenvironment at least during the first generation. However, the limitations include (i) a high proportion (60%) of MPM do not grow as PDX; (ii) the tumor microenvironment is replaced by murine cells over generations; and (iii) the immune context is modified, which is not suitable for evaluation of immunotherapeutic strategies (95, 97). PDX also requires access to tumor samples, which is not easy in the case of a relatively rare cancer as MM.

The use of a humanized mouse model of MM might be a good alternative to study the anti-tumor immune response. In these models, the mouse immune system is replaced by a human immune system. NSG mice are used for this research; they are deficient in the interleukin 2 receptor gamma subunit (IL-2Rγ) that is involved in differentiation and function of many hematopoietic cells (98). This feature confers a great advantage to study immunotherapy strategies in an environment that closely resembles human patients. However, these models present some limits, including the cost, the time to obtain NSG mice reconstituted with a human immune system, the risk of an incomplete differentiation of haematopoietic stem cells, and the graft vs. host reaction, which could limit the duration of the experiments.

### Spheroid Models

In order to overcome some defaults of the existing *in vivo* models, 3D tumor spheroids, positioned between 2D cell culture and animal models, have been developed (99). MCTS involves culturing tumor cells in non-adherent conditions to obtain well-rounded cellular structures after 48–72 h. This culture mode has been applied to MM cell lines. The 3D organization of cells induces major changes in gene expression compared to 2D culture (100, 101). Indeed, some pathways involved in resistance to cell death are differentially regulated in monolayer and MCTS, and thus these models better mimic resistance to treatment compared with monolayer cells (64, 102–106). These models also reproduce the diffusion constraint of therapeutic molecules, such as antibodies or nanovectors (106–109). The 3D structures also share common features with tumors from patients (101). This aspect has been notably demonstrated in the field of autophagy (110–112). Indeed, resistance to treatment is associated with autophagy in MCTS and tumors *in situ*.

The main weakness of the current 3D models is the absence of cells from the microenvironment. An alternative to MCTS is the use of tumoroids, which include tumor cells and infiltrated cells (99, 113). However, these models require access to fresh surgical MM samples. Co-cultures serve as an alternative. MCTS of non-small-cell lung cancer, pancreatic, and breast cancer tumor cells supplemented with fibroblasts and/or macrophages have been described (114–116). The tumor-associated macrophages (TAM) obtained in these models present similar characteristics to those observed in tumors, namely increased resistance to treatment and an improved cytokine environment. These aspects are crucial for immunology studies. MCTS that include different cell types might constitute interesting tools for preliminary studies. They are achieved by combining stromal and immune cells issued from cell lines or isolated from different donors. However, although their relevance needs to be confirmed, MCTS reproduce a partial human microenvironment that is completely absent from cell-derived xenograft.

## Conclusion

Multiple classical preclinical models of cancer have been applied, and new ones are under development, to test the potential effect of anti-cancer drugs on human MM. Each available model has benefits and limits (Table 1), and they must be selected depending on the objectives of the experiment. Overall, these models incompletely reproduce human MM, given that they do not consider the anatomical or biological tumor microenvironment or the intra-tumor heterogeneity of the tumors. The immunological context is not fully reconstructed, even with humanized mice. Three-dimensional spheroid cultures that have been developed as *in vitro* systems, and co-cultures with immune and stromal cells should be considered to improve the relevance of these models. Inter-tumor heterogeneity is also insufficiently studied because most models proceed with MM cell lines or tumors not always characterized at the molecular level, especially concerning the mutation burden and chromosomal abnormalities of the tumor cells. These models remain surrogates; however, they are of paramount importance in translational research and this encourage new developments to improve their predictability. Among recent developments are PDX models and the generation of GEM that carry mutations identified in human MM tumor cells. These approaches may be useful, but PDX and GEM models in general are complex and have limitations in the immune environment and animal cost. Their application in the context of MM heterogeneity will require the use of multiple cell lines according to their molecular profile. To achieve sound results with significant statistical value, including kinetics and dose-effect relationships, a large number of animals would also be needed, unless solid ancillary results are available. Besides economic issues, the 3R rules (Replacement, Reduction, and Refinemen) on the use of animals in scientific procedures are recommended. The identification of biomarkers to follow tumor evolution in response to anti-cancer drugs is of importance to limit the number of animals (117). Lower attrition rates for oncology drugs would be obtained with more predictive models (72, 104, 117, 118). Consequently, it is necessary to develop alternatives for replacement, robust and reproducible bases for reduction, and the use of advanced technologies for refinement (119).

Appropriately designed and analyzed preclinical assays are required (72), with the aim to identify new anti-cancer compounds for MM and novel biomarkers for sensitivity or resistance, which are essential to predict the tumor response. Although animal models are considered to be the most relevant, the development of sophisticated *in vitro* multicellular models should be encouraged. The continuing increase in the knowledge about mesothelial carcinogenesis will permit the use of more pertinent cell models that represent the MM tumor. New approaches not yet used in MM should be explored, including organ-on-a-chip technologies or *in silico* biological systems using computational modeling and machine learning (120, 121). Powerful technological tools should allow researchers to establish models with MM cells that grow in a more accurate tumor microenvironment, and possible *in situ* molecular analyses of tumor cells. The use of well-characterized tumor cells, classified in subgroups of molecular classifications or characterized by histo-molecular gradients, is particularly important regarding the molecular heterogeneity of human MM. This endeavor should allow researchers to obtain representative results of a given type of tumors.

The ongoing preclinical models should be improved with regard to precision, reproducibility, and predictivity, and the results should be supported by different approaches. Some standardization might be helpful. The use of existing consortia and/or the development of new consortia will allow the inclusion of more tumor samples in studies and increase the number of relevant cell models. These factors will enable researchers to adequately cover mesothelioma heterogeneity and be able to afford the high costs of new technologies. Some authors have recommended improving the reliability of preclinical cancer studies by using detailed information on the experimental methodology, different approaches, the publication of negative data, and better dialogue between physicians and scientists (122, 123). These factors are particularly important within the actual context of precision medicine, which implements complex methodologies and multidisciplinary investigations and has a high cost.

## Author Contributions

M-CJ and CB prepared the figures. CB, M-CJ, and DJ wrote the review.

### Conflict of Interest

The authors declare that the research was conducted in the absence of any commercial or financial relationships that could be construed as a potential conflict of interest.

## Abbreviations

2D: two dimensional
3D: three dimensional
CDX: cell-derived xenograft
CGH: comparative genomic hybridization
GEM: genetically modified mice
hom: homozygous
htz: heterozygous
IPl: intra-pleural
IPe: intra-peritoneal
luc: firefly luciferase
MCTS: multicellular tumor spheroids
MM: malignant mesothelioma
MPM: malignant pleural mesothelioma
MPeM: malignant peritoneal mesothelioma
MRI: magnetic resonance imaging
NGS: next generation sequencing
NSG: NOD-scid IL2Rγnull
PDX: patient-derived xenografts
PMTX: pemetrexed
SC: subcutaneous
SNP: single nucleotide polymorphism
SPECT: single photon emission computed tomography
TSG: tumor suppressor genes.

## References

[1] Zalcman G Mazieres J Margery J Greillier L Audigier-Valette C Moro-Sibilot D. Bevacizumab for newly diagnosed pleural mesothelioma in the mesothelioma avastin cisplatin pemetrexed study (MAPS): a randomised, controlled, open-label, phase 3 trial. Lancet. (2016) 387:1405–14. 10.1016/S0140-6736(15)01238-6 26719230

[2] Calabro L Morra A Fonsatti E Cutaia O Amato G Giannarelli D. Tremelimumab for patients with chemotherapy-resistant advanced malignant mesothelioma: an open-label, single-arm, phase 2 trial. Lancet Oncol. (2013) 14:1104–11. 10.1016/S1470-2045(13)70381-4 24035405

[3] Maio M Scherpereel A Calabro L Aerts J Cedres Perez S Bearz A. Tremelimumab as second-line or third-line treatment in relapsed malignant mesothelioma (DETERMINE): a multicentre, international, randomised, double-blind, placebo-controlled phase 2b trial. Lancet Oncol. (2017) 18:1261–73. 10.1016/S1470-2045(17)30446-1 28729154

[4] Scherpereel A Mazieres J Greillier L Lantuejoul S Do P Bylicki O. Nivolumab or nivolumab plus ipilimumab in patients with relapsed malignant pleural mesothelioma (IFCT-1501 MAPS2): a multicentre, open-label, randomised, non-comparative, phase 2 trial. Lancet Oncol. (2019) 20:239–53. 10.1016/S1470-2045(18)30765-4 30660609

[5] Adusumilli PS Cherkassky L Villena-Vargas J Colovos C Servais E Plotkin J. Regional delivery of mesothelin-targeted CAR T cell therapy generates potent and long-lasting CD4-dependent tumor immunity. Sci Transl Med. (2014) 6:261ra151. 10.1126/scitranslmed.3010162 25378643 PMC4373413

[6] Aerts J de Goeje PL Cornelissen R Kaijen-Lambers MEH Bezemer K van der Leest CH. Autologous dendritic cells pulsed with allogeneic tumor cell lysate in mesothelioma: from mouse to human. Clin Cancer Res. (2018) 24:766–76. 10.1158/1078-0432.CCR-17-2522 29233904

[7] Beatty GL Haas AR Maus MV Torigian DA Soulen MC Plesa G. Mesothelin-specific chimeric antigen receptor mRNA-engineered T cells induce anti-tumor activity in solid malignancies. Cancer Immunol Res. (2014) 2:112–20. 10.1158/2326-6066.CIR-13-0170 24579088 PMC3932715

[8] Belderbos RA Baas P Berardi R Cornelissen R Fennell DA van Meerbeeck JP. A multicenter, randomized, phase II/III study of dendritic cells loaded with allogeneic tumor cell lysate (MesoPher) in subjects with mesothelioma as maintenance therapy after chemotherapy: DENdritic cell immunotherapy for mesothelioma (DENIM) trial. Transl Lung Cancer Res. (2019) 8:280–5. 10.21037/tlcr.2019.05.05 31367541 PMC6626859

[9] Cornelissen R Hegmans JP Maat AP Kaijen-Lambers ME Bezemer K Hendriks RW. Extended tumor control after dendritic cell vaccination with low-dose cyclophosphamide as adjuvant treatment in patients with malignant pleural mesothelioma. Am J Respir Crit Care Med. (2016) 193:1023–31. 10.1164/rccm.201508-1573OC 26652184

[10] Hegmans JP Veltman JD Lambers ME de Vries IJ Figdor CG Hendriks RW. Consolidative dendritic cell-based immunotherapy elicits cytotoxicity against malignant mesothelioma. Am J Respir Crit Care Med. (2010) 181:1383–90. 10.1164/rccm.200909-1465OC 20167848

[11] Hung YP Dong F Watkins JC Nardi V Bueno R Dal Cin P. Identification of ALK rearrangements in malignant peritoneal mesothelioma. JAMA Oncol. (2018) 4:235–8. 10.1001/jamaoncol.2017.2918 28910456 PMC5838580

[12] Joseph NM Chen YY Nasr A Yeh I Talevich E Onodera C. Genomic profiling of malignant peritoneal mesothelioma reveals recurrent alterations in epigenetic regulatory genes BAP1, SETD2, and DDX3X. Mod Pathol. (2017) 30:246–54. 10.1038/modpathol.2016.188 27813512 PMC5288276

[13] Shrestha R Nabavi N Lin YY Mo F Anderson S Volik S. BAP1 haploinsufficiency predicts a distinct immunogenic class of malignant peritoneal mesothelioma. Genome Med. (2019) 11:8. 10.1186/s13073-019-0620-3 30777124 PMC6378747

[14] Bueno R Stawiski EW Goldstein LD Durinck S De Rienzo A Modrusan Z. Comprehensive genomic analysis of malignant pleural mesothelioma identifies recurrent mutations, gene fusions and splicing alterations. Nat Genet. (2016) 48:407–16. 10.1038/ng.3520 26928227

[15] Jean D Daubriac J Le Pimpec-Barthes F Galateau-Salle F Jaurand MC. Molecular changes in mesothelioma with an impact on prognosis and treatment. Arch Pathol Lab Med. (2012) 136:277–93. 10.5858/arpa.2011-0215-RA 22372904

[16] Yoshikawa Y Emi M Hashimoto-Tamaoki T Ohmuraya M Sato A Tsujimura T. High-density array-CGH with targeted NGS unmask multiple noncontiguous minute deletions on chromosome 3p21 in mesothelioma. Proc Natl Acad Sci USA. (2016) 113:13432–7. 10.1073/pnas.1612074113 27834213 PMC5127333

[17] Mansfield AS Peikert T Smadbeck JB Udell JBM Garcia-Rivera E Elsbernd L. Neoantigenic potential of complex chromosomal rearrangements in mesothelioma. J Thorac Oncol. (2019) 14:276–87. 10.1016/j.jtho.2018.10.001 30316012 PMC6348045

[18] Hmeljak J Sanchez-Vega F Hoadley KA Shih J Stewart C Heiman D. Integrative molecular characterization of malignant pleural mesothelioma. Cancer Discov. (2018) 8:1548–65. 10.1158/2159-8290.CD-18-0804 30322867 PMC6310008

[19] Tallet A Nault JC Renier A Hysi I Galateau-Salle F Cazes A. Overexpression and promoter mutation of the TERT gene in malignant pleural mesothelioma. Oncogene. (2014) 33:3748–52. 10.1038/onc.2013.351 23975423

[20] Betti M Casalone E Ferrante D Aspesi A Morleo G Biasi A. Germline mutations in DNA repair genes predispose asbestos-exposed patients to malignant pleural mesothelioma. Cancer Lett. (2017) 405:38–45. 10.1016/j.canlet.2017.06.028 28687356

[21] Hassan R Morrow B Thomas A Walsh T Lee MK Gulsuner S. Inherited predisposition to malignant mesothelioma and overall survival following platinum chemotherapy. Proc Natl Acad Sci USA. (2019) 116:9008–13. 10.1073/pnas.1821510116 30975761 PMC6500142

[22] Panou V Gadiraju M Wolin A Weipert CM Skarda E Husain AN. Frequency of germline mutations in cancer susceptibility genes in malignant mesothelioma. J Clin Oncol. (2018) 36:2863–71. 10.1200/JCO.2018.36.15_suppl.8564 30113886 PMC6804864

[23] Pastorino S Yoshikawa Y Pass HI Emi M Nasu M Pagano I A subset of mesotheliomas with improved survival occurring in carriers of BAP1 and other germline mutations. J Clin Oncol. (2018) 36:3485–94. 10.1200/JCO.2018.79.0352 PMC7162737 30376426

[24] Testa JR Cheung M Pei J Below JE Tan Y Sementino E. Germline BAP1 mutations predispose to malignant mesothelioma. Nat Genet. (2011) 43:1022–5. 10.1038/ng.912 21874000 PMC3184199

[25] Christensen BC Houseman EA Godleski JJ Marsit CJ Longacker JL Roelofs CR. Epigenetic profiles distinguish pleural mesothelioma from normal pleura and predict lung asbestos burden and clinical outcome. Cancer Res. (2009) 69:227–34. 10.1158/0008-5472.CAN-08-2586 19118007 PMC2744125

[26] Goto Y Shinjo K Kondo Y Shen L Toyota M Suzuki H. Epigenetic profiles distinguish malignant pleural mesothelioma from lung adenocarcinoma. Cancer Res. (2009) 69:9073–82. 10.1158/0008-5472.CAN-09-1595 19887624

[27] McLoughlin KC Kaufman AS Schrump DS. Targeting the epigenome in malignant pleural mesothelioma. Transl Lung Cancer Res. (2017) 6:350–65. 10.21037/tlcr.2017.06.06 28713680 PMC5504118

[28] LaFave LM Beguelin W Koche R Teater M Spitzer B Chramiec A. Loss of BAP1 function leads to EZH2-dependent transformation. Nat Med. (2015) 21:1344–9. 10.1038/nm.3947 26437366 PMC4636469

[29] Felley-Bosco E Rehrauer H. Non-coding transcript heterogeneity in mesothelioma: insights from asbestos-exposed mice. Int J Mol Sci. (2018) 19:1163. 10.3390/ijms19041163 29641489 PMC5979355

[30] Reid G. MicroRNAs in mesothelioma: from tumour suppressors and biomarkers to therapeutic targets. J Thorac Dis. (2015) 7:1031–40. 10.3978/j.issn.2072-1439.2015.04.56 26150916 PMC4466421

[31] Singh AS Heery R Gray SG. *In silico* and *in vitro* analyses of LncRNAs as potential regulators in the transition from the epithelioid to sarcomatoid histotype of malignant pleural mesothelioma (MPM). Int J Mol Sci. (2018) 19:1297. 10.3390/ijms19051297 29701689 PMC5983793

[32] Jaurand MC Jean D Biomolecular pathways and malignant pleural mesothelioma. In: Mineo CT, editor. Malignant Pleural Mesothelioma: Present Status and Future Directions. Sharjah: Bentham Science Publishers Ltd (2016). p. 169–92.

[33] Yap TA Aerts JG Popat S Fennell DA. Novel insights into mesothelioma biology and implications for therapy. Nat Rev Cancer. (2017) 17:475–88. 10.1038/nrc.2017.42 28740119

[34] Husain AN Colby T Ordonez N Krausz T Attanoos R Beasley MB. Guidelines for pathologic diagnosis of malignant mesothelioma: 2012 update of the consensus statement from the international mesothelioma interest group. Arch Pathol Lab Med. (2013) 137:647–67. 10.5858/arpa.2012-0214-OA 22929121

[35] Oehl K Vrugt B Opitz I Meerang M. Heterogeneity in Malignant Pleural Mesothelioma. Int J Mol Sci. (2018) 19. 10.3390/ijms19061603 29848954 PMC6032160

[36] de Reynies A Jaurand MC Renier A Couchy G Hysi I Elarouci N. Molecular classification of malignant pleural mesothelioma: identification of a poor prognosis subgroup linked to the epithelial-to-mesenchymal transition. Clin Cancer Res. (2014) 20:1323–34. 10.1158/1078-0432.CCR-13-2429 24443521

[37] Tranchant R Quetel L Tallet A Meiller C Renier A de Koning L. Co-occurring mutations of tumor suppressor genes, LATS2 and NF2, in malignant pleural mesothelioma. Clin Cancer Res. (2017) 23:3191–202. 10.1158/1078-0432.CCR-16-1971 28003305

[38] Blum Y Meiller C Quetel L Elarouci N Ayadi M Tashtanbaeva D. Dissecting heterogeneity in malignant pleural mesothelioma through histo-molecular gradients for clinical applications. Nat Commun. (2019) 10:1333. 10.1038/s41467-019-09307-6 30902996 PMC6430832

[39] Blum Y Jaurand MC De Reynies A Jean D. Unraveling the cellular heterogeneity of malignant pleural mesothelioma through a deconvolution approach. Mol Cell Oncol. (2019) 6:1610322. 10.1080/23723556.2019.1610322 31211240 PMC6548486

[40] Alcala N Mangiante L Le-Stang N Gustafson CE Boyault S Damiola F. Redefining malignant pleural mesothelioma types as a continuum uncovers immune-vascular interactions. EBioMedicine. (2019) 48:191–202. 10.1016/j.ebiom.2019.09.003 31648983 PMC6838392

[41] Comertpay S Pastorino S Tanji M Mezzapelle R Strianese O Napolitano A. Evaluation of clonal origin of malignant mesothelioma. J Transl Med. (2014) 12:301. 10.1186/s12967-014-0301-3 25471750 PMC4255423

[42] Oey H Daniels M Relan V Chee TM Davidson MR Yang IA. Whole-genome sequencing of human malignant mesothelioma tumours and cell lines. Carcinogenesis. (2019) 40:724–34. 10.1093/carcin/bgz066 31038674

[43] Minnema-Luiting J Vroman H Aerts J Cornelissen R. Heterogeneity in immune cell content in malignant pleural mesothelioma. Int J Mol Sci. (2018) 19:1041. 10.3390/ijms19041041 29601534 PMC5979422

[44] Salaroglio IC Kopecka J Napoli F Pradotto M Maletta F Costardi L. Potential diagnostic and prognostic role of microenvironment in malignant pleural mesothelioma. J Thorac Oncol. (2019) 14:1458–71. 10.1016/j.jtho.2019.03.029 31078776

[45] Kiyotani K Park JH Inoue H Husain A Olugbile S Zewde M. Integrated analysis of somatic mutations and immune microenvironment in malignant pleural mesothelioma. Oncoimmunology. (2017) 6:e1278330. 10.1080/2162402X.2016.1278330 28344893 PMC5353938

[46] Maley CC Aktipis A Graham TA Sottoriva A Boddy AM Janiszewska M. Classifying the evolutionary and ecological features of neoplasms. Nat Rev Cancer. (2017) 17:605–19. 10.1038/nrc.2017.69 28912577 PMC5811185

[47] Mutti L Peikert T Robinson BWS Scherpereel A Tsao AS de Perrot M. Scientific advances and new frontiers in mesothelioma therapeutics. J Thorac Oncol. (2018) 13:1269–83. 10.1016/j.jtho.2018.06.011 29966799 PMC6643278

[48] Tagawa M Tada Y Shimada H Hiroshima K. Gene therapy for malignant mesothelioma: current prospects and challenges. Cancer Gene Ther. (2013) 20:150–6. 10.1038/cgt.2013.1 23392201

[49] Abu Lila AS Kato C Fukushima M Huang CL Wada H Ishida T. Downregulation of thymidylate synthase by RNAi molecules enhances the antitumor effect of pemetrexed in an orthotopic malignant mesothelioma xenograft mouse model. Int J Oncol. (2016) 48:1399–407. 10.3892/ijo.2016.3367 26847426

[50] Favoni RE Daga A Malatesta P Florio T. Preclinical studies identify novel targeted pharmacological strategies for treatment of human malignant pleural mesothelioma. Br J Pharmacol. (2012) 166:532–53. 10.1111/j.1476-5381.2012.01873.x 22289125 PMC3417486

[51] Giovannetti E Zucali PA Assaraf YG Leon LG Smid K Alecci C. Preclinical emergence of vandetanib as a potent antitumour agent in mesothelioma: molecular mechanisms underlying its synergistic interaction with pemetrexed and carboplatin. Br J Cancer. (2011) 105:1542–53. 10.1038/bjc.2011.400 21970874 PMC3242521

[52] Hanauske AR. The role of alimta in the treatment of malignant pleural mesothelioma: an overview of preclinical and clinical trials. Lung Cancer. (2004) 45(Suppl. 1):S121–4. 10.1016/j.lungcan.2004.04.021 15261444

[53] Iwahori K Serada S Fujimoto M Ripley B Nomura S Mizuguchi H. SOCS-1 gene delivery cooperates with cisplatin plus pemetrexed to exhibit preclinical antitumor activity against malignant pleural mesothelioma. Int J Cancer. (2013) 132:459–71. 10.1002/ijc.27611 22532243

[54] Leon LG Gemelli M Sciarrillo R Avan A Funel N Giovannetti E. Synergistic activity of the c-Met and tubulin inhibitor tivantinib (ARQ197) with pemetrexed in mesothelioma cells. Curr Drug Targets. (2014) 15:1331–40. 10.2174/1389450116666141205160924 25483224

[55] Ueno T Toyooka S Fukazawa T Kubo T Soh J Asano H. Preclinical evaluation of microRNA-34b/c delivery for malignant pleural mesothelioma. Acta Med Okayama. (2014) 68:23–6. 10.18926/AMO/52140 24553485

[56] Ando H Kobayashi S Abu Lila AS Eldin NE Kato C Shimizu T. Advanced therapeutic approach for the treatment of malignant pleural mesothelioma via the intrapleural administration of liposomal pemetrexed. J Control Release. (2015) 220:29–36. 10.1016/j.jconrel.2015.10.019 26476173

[57] Meerang M Boss A Kenkel D Broggini-Tenzer A Berard K Lauk O. Evaluation of imaging techniques for the assessment of tumour progression in an orthotopic rat model of malignant pleural mesotheliomadagger. Eur J Cardiothorac Surg. (2015) 47:e34–41. 10.1093/ejcts/ezu393 25344922

[58] Iochmann S Lerondel S Blechet C Lavergne M Pesnel S Sobilo J. Monitoring of tumour progression using bioluminescence imaging and computed tomography scanning in a nude mouse orthotopic model of human small cell lung cancer. Lung Cancer. (2012) 77:70–6. 10.1016/j.lungcan.2012.01.009 22321610

[59] Nayak TK Bernardo M Milenic DE Choyke PL Brechbiel MW. Orthotopic pleural mesothelioma in mice: SPECT/CT and MR imaging with HER1- and HER2-targeted radiolabeled antibodies. Radiology. (2013) 267:173–82. 10.1148/radiol.12121021 23329660 PMC3606545

[60] Behbehani AM Hunter WJ Chapman AL Lin F. Studies of a human mesothelioma. Hum Pathol. (1982) 13:862–6. 10.1016/S0046-8177(82)80083-X 7106748

[61] Reale FR Griffin TW Compton JM Graham S Townes PL Bogden A. Characterization of a human malignant mesothelioma cell line (H-MESO-1): a biphasic solid and ascitic tumor model. Cancer Res. (1987) 47:3199–205. 3555770

[62] Gueugnon F Leclercq S Blanquart C Sagan C Cellerin L Padieu M. Identification of novel markers for the diagnosis of malignant pleural mesothelioma. Am J Pathol. (2011) 178:1033–42. 10.1016/j.ajpath.2010.12.014 21356356 PMC3070574

[63] Jean D Thomas E Manie E Renier A de Reynies A Lecomte C. Syntenic relationships between genomic profiles of fiber-induced murine and human malignant mesothelioma. Am J Pathol. (2011) 178:881–94. 10.1016/j.ajpath.2010.10.039 21281820 PMC3070549

[64] Tranchant R Quetel L Montagne F De Wolf J Meiller C De Koning L. Assessment of signaling pathway inhibitors and identification of predictive biomarkers in malignant pleural mesothelioma. Lung Cancer. (2018) 126:15–24. 10.1016/j.lungcan.2018.10.015 30527180

[65] Zeng L Buard A Monnet I Boutin C Fleury J Saint-Etienne L. *In vitro* effects of recombinant human interferon gamma on human mesothelioma cell lines. Int J Cancer. (1993) 55:515–20. 10.1002/ijc.2910550331 8375935

[66] Zeng L Fleury-Feith J Monnet I Boutin C Bignon J Jaurand MC. Immunocytochemical characterization of cell lines from human malignant mesothelioma: characterization of human mesothelioma cell lines by immunocytochemistry with a panel of monoclonal antibodies. Hum Pathol. (1994) 25:227–34. 10.1016/0046-8177(94)90192-9 7512071

[67] Chernova T Sun XM Powley IR Galavotti S Grosso S Murphy FA. Molecular profiling reveals primary mesothelioma cell lines recapitulate human disease. Cell Death Differ. (2016) 23:1152–64. 10.1038/cdd.2015.165 26891694 PMC4946883

[68] Smeele P d'Almeida SM Meiller C Chene AL Liddell C Cellerin L. Brain-derived neurotrophic factor, a new soluble biomarker for malignant pleural mesothelioma involved in angiogenesis. Mol Cancer. (2018) 17:148. 10.1186/s12943-018-0891-0 30309369 PMC6180566

[69] Jongsma J van Montfort E Vooijs M Zevenhoven J Krimpenfort P van der Valk M. A conditional mouse model for malignant mesothelioma. Cancer Cell. (2008) 13:261–71. 10.1016/j.ccr.2008.01.030 18328429

[70] Jean D Jaurand MC. Mesotheliomas in Genetically engineered mice unravel mechanism of mesothelial carcinogenesis. Int J Mol Sci. (2018) 19:2191. 10.3390/ijms19082191 30060470 PMC6121615

[71] Kukuyan AM Sementino E Kadariya Y Menges CW Cheung M Tan Y. Inactivation of Bap1 cooperates with losses of Nf2 and Cdkn2a to drive the development of pleural malignant mesothelioma in conditional mouse models. Cancer Res. (2019) 79:4113–23. 10.1158/0008-5472.CAN-18-4093 31151962 PMC6697648

[72] Ruggeri BA Camp F Miknyoczki S. Animal models of disease: pre-clinical animal models of cancer and their applications and utility in drug discovery. Biochem Pharmacol. (2014) 87:150–61. 10.1016/j.bcp.2013.06.020 23817077

[73] Singh M Murriel CL Johnson L. Genetically engineered mouse models: closing the gap between preclinical data and trial outcomes. Cancer Res. (2012) 72:2695–700. 10.1158/0008-5472.CAN-11-2786 22593194

[74] Disselhorst MJ Quispel-Janssen J Lalezari F Monkhorst K de Vries JF van der Noort V. Ipilimumab and nivolumab in the treatment of recurrent malignant pleural mesothelioma (INITIATE): results of a prospective, single-arm, phase 2 trial. Lancet Respir Med. (2019) 7:260–70. 10.1016/S2213-2600(18)30420-X 30660511

[75] Fennell DA Kirkpatrick E Cozens K Nye M Lester J Hanna G. CONFIRM: a double-blind, placebo-controlled phase III clinical trial investigating the effect of nivolumab in patients with relapsed mesothelioma: study protocol for a randomised controlled trial. Trials. (2018) 19:233. 10.1186/s13063-018-2602-y 29669604 PMC5907297

[76] Mansfield AS Zauderer MG. Nivo-lution in mesothelioma. Clin Cancer Res. (2019) 25:5438–40. 10.1158/1078-0432.CCR-19-1836 31315884 PMC6744981

[77] Moalli PA MacDonald JL Goodglick LA Kane AB. Acute injury and regeneration of the mesothelium in response to asbestos fibers. Am J Pathol. (1987) 128:426–45. 2820232 PMC1899662

[78] Jackaman C Bundell CS Kinnear BF Smith AM Filion P van Hagen D. IL-2 intratumoral immunotherapy enhances CD8+ T cells that mediate destruction of tumor cells and tumor-associated vasculature: a novel mechanism for IL-2. J Immunol. (2003) 171:5051–63. 10.4049/jimmunol.171.10.5051 14607902

[79] Davis MR Manning LS Whitaker D Garlepp MJ Robinson BW. Establishment of a murine model of malignant mesothelioma. Int J Cancer. (1992) 52:881–6. 10.1002/ijc.2910520609 1459729

[80] Craighead JE Akley NJ Gould LB Libbus BL. Characteristics of tumors and tumor cells cultured from experimental asbestos-induced mesotheliomas in rats. Am J Pathol. (1987) 129:448–62. 2827488 PMC1899808

[81] Roulois D Deshayes S Guilly MN Nader JS Liddell C Robard M. Characterization of preneoplastic and neoplastic rat mesothelial cell lines: the involvement of TETs, DNMTs, and 5-hydroxymethylcytosine. Oncotarget. (2016) 7:34664–87. 10.18632/oncotarget.8970 27129173 PMC5085183

[82] Nader JS Abadie J Deshayes S Boissard A Blandin S Blanquart C. Characterization of increasing stages of invasiveness identifies stromal/cancer cell crosstalk in rat models of mesothelioma. Oncotarget. (2018) 9:16311–29. 10.18632/oncotarget.24632 29662647 PMC5893242

[83] Shi Y Hollenstein A Felley-Bosco E Fraefel C Ackermann M Soltermann A. Bioluminescence imaging for *in vivo* monitoring of local recurrence mesothelioma model. Lung Cancer. (2011) 71:370–1. 10.1016/j.lungcan.2010.12.020 21277037

[84] Crisanti MC Wallace AF Kapoor V Vandermeers F Dowling ML Pereira LP. The HDAC inhibitor panobinostat (LBH589) inhibits mesothelioma and lung cancer cells *in vitro* and *in vivo* with particular efficacy for small cell lung cancer. Mol Cancer Ther. (2009) 8:2221–31. 10.1158/1535-7163.MCT-09-0138 19671764 PMC3605895

[85] Laszlo V Valko Z Ozsvar J Kovacs I Garay T Hoda MA. The FAK inhibitor BI 853520 inhibits spheroid formation and orthotopic tumor growth in malignant pleural mesothelioma. J Mol Med. (2019) 97:231–42. 10.1007/s00109-018-1725-7 30539198 PMC6348072

[86] Pulito C Korita E Sacconi A Valerio M Casadei L Lo Sardo F. Dropwort-induced metabolic reprogramming restrains YAP/TAZ/TEAD oncogenic axis in mesothelioma. J Exp Clin Cancer Res. (2019) 38:349. 10.1186/s13046-019-1352-3 31399037 PMC6689183

[87] Abe S Kaneko MK Tsuchihashi Y Izumi T Ogasawara S Okada N. Antitumor effect of novel anti-podoplanin antibody NZ-12 against malignant pleural mesothelioma in an orthotopic xenograft model. Cancer Sci. (2016) 107:1198–205. 10.1111/cas.12985 27294401 PMC5021042

[88] Golfier S Kopitz C Kahnert A Heisler I Schatz CA Stelte-Ludwig B. Anetumab ravtansine: a novel mesothelin-targeting antibody-drug conjugate cures tumors with heterogeneous target expression favored by bystander effect. Mol Cancer Ther. (2014) 13:1537–48. 10.1158/1535-7163.MCT-13-0926 24714131

[89] Krishnan H Rayes J Miyashita T Ishii G Retzbach EP Sheehan SA. Podoplanin: an emerging cancer biomarker and therapeutic target. Cancer Sci. (2018) 109:1292–9. 10.1111/cas.13580 29575529 PMC5980289

[90] Scales SJ Gupta N Pacheco G Firestein R French DM Koeppen H. An antimesothelin-monomethyl auristatin e conjugate with potent antitumor activity in ovarian, pancreatic, and mesothelioma models. Mol Cancer Ther. (2014) 13:2630–40. 10.1158/1535-7163.MCT-14-0487-T 25249555

[91] Zhang YF Phung Y Gao W Kawa S Hassan R Pastan I. New high affinity monoclonal antibodies recognize non-overlapping epitopes on mesothelin for monitoring and treating mesothelioma. Sci Rep. (2015) 5:9928. 10.1038/srep09928 25996440 PMC4440525

[92] Zhang J Khanna S Jiang Q Alewine C Miettinen M Pastan I. Efficacy of anti-mesothelin immunotoxin RG7787 plus nab-paclitaxel against mesothelioma patient-derived xenografts and mesothelin as a biomarker of tumor response. Clin Cancer Res. (2017) 23:1564–74. 10.1158/1078-0432.CCR-16-1667 27635089 PMC5352557

[93] Moon EK Carpenito C Sun J Wang LC Kapoor V Predina J. Expression of a functional CCR2 receptor enhances tumor localization and tumor eradication by retargeted human T cells expressing a mesothelin-specific chimeric antibody receptor. Clin Cancer Res. (2011) 17:4719–30. 10.1158/1078-0432.CCR-11-0351 21610146 PMC3612507

[94] Zhao Y Moon E Carpenito C Paulos CM Liu X Brennan AL. Multiple injections of electroporated autologous T cells expressing a chimeric antigen receptor mediate regression of human disseminated tumor. Cancer Res. (2010) 70:9053–61. 10.1158/0008-5472.CAN-10-2880 20926399 PMC2982929

[95] Jung J Seol HS Chang S. The generation and application of patient-derived xenograft model for cancer research. Cancer Res Treat. (2018) 50:1–10. 10.4143/crt.2017.307 28903551 PMC5784646

[96] Chahinian AP Beranek JT Suzuki Y Bekesi JG Wisniewski L Selikoff IJ. Transplantation of human malignant mesothelioma into nude mice. Cancer Res. (1980) 40:181–5. 7349896

[97] Wu L Allo G John T Li M Tagawa T Opitz I. Patient-derived xenograft establishment from human malignant pleural mesothelioma. Clin Cancer Res. (2017) 23:1060–7. 10.1158/1078-0432.CCR-16-0844 27683181

[98] Cao X Shores EW Hu-Li J Anver MR Kelsall BL Russell SM. Defective lymphoid development in mice lacking expression of the common cytokine receptor gamma chain. Immunity. (1995) 2:223–38. 10.1016/1074-7613(95)90047-0 7697543

[99] Weiswald LB Bellet D Dangles-Marie V. Spherical cancer models in tumor biology. Neoplasia. (2015) 17:1–15. 10.1016/j.neo.2014.12.004 25622895 PMC4309685

[100] Kim H Phung Y Ho M. Changes in global gene expression associated with 3D structure of tumors: an *ex vivo* matrix-free mesothelioma spheroid model. PLoS ONE. (2012) 7:e39556. 10.1371/journal.pone.0039556 22737246 PMC3380922

[101] Barbone D Van Dam L Follo C Jithesh PV Zhang SD Richards WG. Analysis of gene expression in 3D spheroids highlights a survival role for ASS1 in mesothelioma. PLoS ONE. (2016) 11:e0150044. 10.1371/journal.pone.0150044 26982031 PMC4794185

[102] Barbone D Yang TM Morgan JR Gaudino G Broaddus VC. Mammalian target of rapamycin contributes to the acquired apoptotic resistance of human mesothelioma multicellular spheroids. J Biol Chem. (2008) 283:13021–30. 10.1074/jbc.M709698200 18339627 PMC2442321

[103] Barbone D Cheung P Battula S Busacca S Gray SG Longley DB. Vorinostat eliminates multicellular resistance of mesothelioma 3D spheroids via restoration of noxa expression. PLoS ONE. (2012) 7:e52753. 10.1371/journal.pone.0052753 23300762 PMC3530471

[104] Daubriac J Fleury-Feith J Kheuang L Galipon J Saint-Albin A Renier A. Malignant pleural mesothelioma cells resist anoikis as quiescent pluricellular aggregates. Cell Death Differ. (2009) 16:1146–55. 10.1038/cdd.2009.32 19343038

[105] Gerogianni I Pitaraki E Jagirdar RM Kouliou O Giannakou L Giannopoulos S 2-Deoxy-glucose enhances the effect of cisplatin and pemetrexed in reducing malignant pleural mesothelioma cell proliferation but not spheroid growth. Anticancer Res. (2019) 39:3809–14. 10.21873/anticanres.13530 31262908

[106] Lei H Hofferberth SC Liu R Colby A Tevis KM Catalano P. Paclitaxel-loaded expansile nanoparticles enhance chemotherapeutic drug delivery in mesothelioma 3-dimensional multicellular spheroids. J Thorac Cardiovasc Surg. (2015) 149:1417–24. 10.1016/j.jtcvs.2015.02.020 25841659

[107] Linot C Poly J Boucard J Pouliquen D Nedellec S Hulin P. PEGylated anionic magnetofluorescent nanoassemblies: impact of their interface structure on magnetic resonance imaging contrast and cellular uptake. ACS Appl Mater Interfaces. (2017) 9:14242–57. 10.1021/acsami.7b01737 28379690

[108] Phung YT Barbone D Broaddus VC Ho M. Rapid generation of *in vitro* multicellular spheroids for the study of monoclonal antibody therapy. J Cancer. (2011) 2:507–14. 10.7150/jca.2.507 22043235 PMC3204399

[109] Xiang X Phung Y Feng M Nagashima K Zhang J Broaddus VC. The development and characterization of a human mesothelioma *in vitro* 3D model to investigate immunotoxin therapy. PLoS ONE. (2011) 6:e14640. 10.1371/journal.pone.0014640 21305058 PMC3031536

[110] Barbone D Ryan JA Kolhatkar N Chacko AD Jablons DM Sugarbaker DJ. The Bcl-2 repertoire of mesothelioma spheroids underlies acquired apoptotic multicellular resistance. Cell Death Dis. (2011) 2:e174. 10.1038/cddis.2011.58 21697949 PMC3169000

[111] Echeverry N Ziltener G Barbone D Weder W Stahel RA Broaddus VC. Inhibition of autophagy sensitizes malignant pleural mesothelioma cells to dual PI3K/mTOR inhibitors. Cell Death Dis. (2015) 6:e1757. 10.1038/cddis.2015.124 25950487 PMC4669703

[112] Follo C Barbone D Richards WG Bueno R Broaddus VC. Autophagy initiation correlates with the autophagic flux in 3D models of mesothelioma and with patient outcome. Autophagy. (2016) 12:1180–94. 10.1080/15548627.2016.1173799 27097020 PMC4990992

[113] Kim KU Wilson SM Abayasiriwardana KS Collins R Fjellbirkeland L Xu Z. A novel *in vitro* model of human mesothelioma for studying tumor biology and apoptotic resistance. Am J Respir Cell Mol Biol. (2005) 33:541–8. 10.1165/rcmb.2004-0355OC 16123394 PMC2715331

[114] Kuen J Darowski D Kluge T Majety M. Pancreatic cancer cell/fibroblast co-culture induces M2 like macrophages that influence therapeutic response in a 3D model. PLoS ONE. (2017) 12:e0182039. 10.1371/journal.pone.0182039 28750018 PMC5531481

[115] Tevis KM Cecchi RJ Colson YL Grinstaff MW. Mimicking the tumor microenvironment to regulate macrophage phenotype and assessing chemotherapeutic efficacy in embedded cancer cell/macrophage spheroid models. Acta Biomater. (2017) 50:271–9. 10.1016/j.actbio.2016.12.037 28011141 PMC5316313

[116] Rebelo SP Pinto C Martins TR Harrer N Estrada MF Loza-Alvarez P. 3D-3-culture: a tool to unveil macrophage plasticity in the tumour microenvironment. Biomaterials. (2018) 163:185–97. 10.1016/j.biomaterials.2018.02.030 29477032

[117] de Bono JS Ashworth A. Translating cancer research into targeted therapeutics. Nature. (2010) 467:543–9. 10.1038/nature09339 20882008

[118] Moreno L Pearson AD. How can attrition rates be reduced in cancer drug discovery? Exp Opin Drug Discov. (2013) 8:363–8. 10.1517/17460441.2013.768984 23373702

[119] Graham ML Prescott MJ. The multifactorial role of the 3Rs in shifting the harm-benefit analysis in animal models of disease. Eur J Pharmacol. (2015) 759:19–29. 10.1016/j.ejphar.2015.03.040 25823812 PMC4441106

[120] Jean-Quartier C Jeanquartier F Jurisica I Holzinger A. *In silico* cancer research towards 3R. BMC Cancer. (2018) 18:408. 10.1186/s12885-018-4302-0 29649981 PMC5897933

[121] Sun W Luo Z Lee J Kim HJ Lee K Tebon P Organ-on-a-chip for cancer and immune organs modeling. Adv Healthc Mater. (2019) 8:e1801363 10.1002/adhm.201801363 30605261 PMC6424124

[122] Begley CG Ellis LM. Drug development: raise standards for preclinical cancer research. Nature. (2012) 483:531–3. 10.1038/483531a 22460880

[123] Kimmelman J Federico C. Consider drug efficacy before first-in-human trials. Nature. (2017) 542:25–7. 10.1038/542025a 28150789

